# Theoretical rejection of fifty-four antineoplastic drugs by different nanofiltration membranes

**DOI:** 10.1007/s11356-023-29830-w

**Published:** 2023-09-19

**Authors:** Teresa I.A. Gouveia, Arminda Alves, Mónica S.F. Santos

**Affiliations:** 1 LEPABE – Laboratory for Process, Environmental, Biotechnology and Energy Engineering, Faculty of Engineering, University of Porto, R. Dr. Roberto Frias, 4200-465 Porto, Portugal; 2 ALiCE – Associate Laboratory in Chemical Engineering, Faculty of Engineering, University of Porto, Rua Dr. Roberto Frias, 4200-465 Porto, Portugal; 3 EPIUnit - Institute of Public Health, University of Porto, Rua das Taipas, no. 135, 4050-600 Porto, Portugal; 4 ITR - Laboratory for Integrative and Translational Research in Population Health, University of Porto, Rua das Taipas, no. 135, 4050-600 Porto, Portugal

**Keywords:** Antineoplastic drugs, Cytostatic drugs, Nanofiltration, Rejection rates, QSAR model

## Abstract

The rise of nanofiltration technologies holds great promise for creating more effective and affordable techniques aiming to remove undesirable pollutants from wastewaters. Despite nanofiltration’s promising potential in removing antineoplastic drugs from liquid matrices, the limited information on this topic makes it important to estimate the rejection rates for a larger number of compounds, particularly the emerging ones, in order to preview the nanofiltration performance. Aiming to have preliminary estimations of the rejection rates of antineoplastic drugs by nanofiltration, 54 antineoplastic drugs were studied in 5 nanofiltration membranes (Desal 5DK, Desal HL, Trisep TS-80, NF270, and NF50), using a quantitative structure-activity relationship (QSAR) model. While this methodology provides useful and reliable predictions of the rejections of compounds by nanofiltration, particularly for hydrophilic and neutral compounds, it is important to note that QSAR results should always be corroborated by experimental assays, as predictions were confirmed to have their limitations (especially for hydrophobic and charged compounds). Out of the 54 studied antineoplastic drugs, 29 were predicted to have a rejection that could go up to 100%, independent of the membrane used. Nonetheless, there were 2 antineoplastic drugs, fluorouracil and thiotepa, for which negligible removals were obtained (<21%). This study’s findings may contribute (i) to the selection of the most appropriate nanofiltration membranes for removing antineoplastic drugs from wastewaters and (ii) to assist in the design of effective treatment approaches for their removal.

**Supplementary Information:**

The online version contains supplementary material available at 10.1007/s11356-023-29830-w.

## Introduction

Antineoplastic drugs, also called anticancer, chemotherapy, chemo, cytotoxic, or hazardous drugs, are pharmaceuticals used in chemotherapy (CDC 2017). One of their main functions is to trigger cellular dysfunction, inhibiting the growth of tumorous cells by changing their metabolism and blocking cell division and reproduction (OSHA 2012). However, the International Agency for Research on Cancer (IARC) concluded that antineoplastic drugs could inhibit tumor growth at the same time as causing it, due to their high toxicity (IARC 1981). Therefore, exposure to these compounds should be avoided whenever possible. Their partial excretion by oncology patients and animals has led to their continuous discharge into the environment, particularly into the water courses due to their low volatility (Cui et al. 2020; Ioele et al. 2022). The presence of antineoplastic drugs in wastewaters and surface waters has been reported worldwide, as long as their inefficient elimination by conventional wastewater treatments currently applied in wastewater treatment plants (WWTPs) (Gouveia et al. 2020; Khasawneh and Palaniandy 2021). Thus, additional treatments to implement in WWTPs, able to effectively remove antineoplastic drugs from wastewaters, is of utmost importance, mitigating their release and accumulation in the environment.

Membrane processes have been confirmed to be promising technologies intending the reduction of micropollutants present in liquid matrices, such as endocrine disrupting compounds, pharmaceutically active compounds, and pesticides (Nghiem et al. 2004; Yangali-Quintanilla et al. 2010). They enclose a high removal efficiency, low energy usage, convenience for scaling up, and the possibility of continuous flow operation (Doménech et al. 2020). Despite nanofiltration possible drawbacks (membrane fouling, generation of a polluted stream, chemical resistance, and limited lifetime of membranes and insufficient rejection of some pollutants in water treatment) (Van der Bruggen et al. 2008), it is a relatively recent advance in membrane technology that usually provides medium to high rejections of compounds present in liquid matrices (Doménech et al. 2020; Nghiem et al. 2004). The need for reliable modeling and simulation tools has also been stated as a limitation associated with the nanofiltration technique (Van der Bruggen et al. 2008).

Up to date, there are hundreds of antineoplastic drugs discovered and approved by the Food and Drug Administration (FDA) (Kinch 2014; Sun et al. 2017). Between them, and up to author’s knowledge, nanofiltration was only applied for 13, being cyclophosphamide the most studied one (mentioned in 6 out of 7 papers) (Cristóvão et al. 2022; Cristóvão et al. 2019; Gouveia et al. 2023; Verliefde et al. 2009; Verliefde et al. 2007a; Wang et al. 2009). On the other side, bicalutamide, cytarabine, fluorouracil, megestrol, mycophenolate mofetil, mycophenolic acid, flutamide, and tamoxifen’s retentions by nanofiltration were reported in only one study each (Cristóvão et al. 2019; Gouveia et al. 2023; Kazner et al. 2008). Regarding the membranes used, Desal 5DK was the most studied one (4 out of 7 studies), tested for tested for bicalutamide, capecitabine, cyclophosphamide, etoposide, flutamide, ifosfamide, megestrol, mycophenolate mofetil, mycophenolic acid, paclitaxel, and tamoxifen (Cristóvão et al. 2022; Cristóvão et al. 2019; Gouveia et al. 2023; Wang et al. 2009). NF50 M10 was the least studied membrane, applied in one study for cytarabine and fluorouracil rejections.

Since this technology has proven to be quite promising in the removal of antineoplastic drugs (with rejections going up to 100%) from liquid matrices, there is a need to obtain/estimate the rejections for a larger number of antineoplastic drugs, particularly the already classified as carcinogenic to humans (such as busulfan, melphalan, or thiotepa), aiming to assist in the design of effective treatment approaches for their removal from wastewaters.

Thus, this study aims to estimate the theoretical rejections of 54 antineoplastic drugs, chosen based on their administration in a Portuguese hospitals and their mechanism of action/classification, using the same nanofiltration membranes that have already been experimentally used for the removal of antineoplastic drugs (Desal 5DK, Desal HL, Trisep TS-80, NF270, and NF50). The theoretical rejections were then compared with experimental data when the information is available. Predictions were based on a quantitative structure-activity relationship (QSAR) model previously developed and validated by Yangali-Quintanilla and co-workers (Yangali-Quintanilla et al. 2010). Cristóvão and co-workers have previously applied the same QSAR model to predict the rejections for 13 antineoplastic drugs (anastrozole, bicalutamide, capecitabine, cyclophosphamide, etoposide, gemcitabine, gefitinib, imatinib, ifosfamide, methotrexate, mycophenolic acid, pazopanib, and tamoxifen) from water, considering a Desal 5DK membrane (Cristóvão et al. 2020). Still, a comparative analysis of the rejection of a higher number of antineoplastic drugs, using different membranes, is still lacking. Thus, this study aims to provide a valuable tool for future research in the field of antineoplastic drug removal from wastewater or surface waters, by predicting the rejection rates of 54 antineoplastic drugs using nanofiltration membranes of different characteristics.

## Characterization and classification of the target antineoplastic drugs

A total of 54 antineoplastic drugs were selected based on (i) their frequency of administration in a Portuguese hospital and (ii) their classification (immunotherapy drugs and enzymes used in chemotherapy were not considered). The chosen antineoplastic drugs are listed in Table 1, as well as some of their physical and chemical properties: molecular weight (MW), pKa, charge at neutral pH, log D, molecular length (*L*), molecular width (*W*), and molecular depth (*D*), which are size properties of each molecule, equivalent width (*eqW*), which is the geometric mean of *W* and *L* and IARC Classification.

**Table 1:** Physico-chemical properties of the target antineoplastic drugs, as well as their IARC classification

	Antineoplastic drug	MW (g/mol)^a^	pKa (strongest basic/acid)^a^	Charge (pH = 7)	Log D^a^	*L* (nm)^b^	*W* (nm)^b^	*D* (nm)^b^	*eqW* (nm)	IARC classification*^,c^
HL-neutral	Azacitidine	244	1.96, 12.55	0	−2.31	0.963	0.671	0.373	0.500	2A
	Bortezomib	384	−0.59, 8.64	0	1.87	1.296	0.838	0.552	0.680	N/A
	Busulfan	246	N/A	0	−0.42	1.251	0.337	0.337	0.337	1
	Capecitabine	359	0.073, 8.63	0	0.39	1.694	0.709	0.535	0.616	N/A
	Carmustine	214	−5.3, 13.36	0	1	0.935	0.438	0.118	0.228	N/A
	Cladribine	286	2.22, 13.89	0	0.01	1.182	0.736	0.460	0.582	N/A
	Clofarabine	304	2.2, 12.71	0	−0.17	1.077	0.597	0.430	0.507	N/A
	Cyclophosphamide	261	0.02, 12.78	0	0.52	0.792	0.552	0.457	0.502	1
	Dactinomycin	1255	−1.1, 10.52	0	−2.38	1.927	1.667	1.545	1.605	N/A
	Decitabine	228	2.09, 13.89	0	−2	1.068	0.433	0.405	0.418	N/A
	Etoposide	589	−3.7, 9.33	0	0.66	1.647	1.300	0.905	1.085	1
	Fludarabine	285	0.76, 12.45	0	−0.86	1.077	0.534	0.455	0.493	N/A
	Fluorouracil	130	−8, 7.18	0	−0.62	0.518	0.518	0.233	0.348	3
	Fotemustine	316	−5.4, 11.81	0	0.84	1.178	0.703	0.627	0.664	N/A
	Ganciclovir	255	0.58, 10.16	0	−1.72	1.129	0.689	0.437	0.548	N/A
	Gemcitabine	263	3.65, 11.52	0	−1.38	0.961	0.493	0.554	0.522	N/A
	Ifosfamide	261	14.64	0	0.68	0.680	0.534	0.524	0.529	N/A
	Melphalan	305	1.29, 9.51	0	−1.05	1.113	0.432	0.346	0.387	1
	Mitomycin C	334	5.76, 14.97	0	−0.51	1.030	0.671	0.602	0.635	2B
	Temozolomide	194	−3.6, 10.46	0	−0.99	0.820	0.450	0.279	0.354	N/A
	Thiotepa	189	−0.3	0	0.48	0.717	0.330	0.284	0.306	1
HL-charged	Bendamustine	358	4.36, 6.41	−1	0.61	1.466	0.446	0.182	0.285	N/A
	Cytarabine	243	4.19, 12.55	−1	−2.2	0.953	0.632	0.440	0.527	N/A
	Dacarbazine	182	1.72, 6.64	−1	0.06	0.852	0.653	0.182	0.345	2B
	Daunorubicin	528	8.01, 10.03	+1	−1.4	1.282	0.903	0.715	0.804	N/A
	Doxorubicin	544	8.01, 10.03	+1	−1.90	1.399	1.155	1.044	1.098	N/A
	Epirubicin	544	8.01, 10.03	+1	−1.89	1.399	0.957	0.903	0.930	N/A
	Antineoplastic drug	MW (g/mol)^a^	pKa (strongest basic/ acid)^a^	Charge (pH=7)	Log D^a^	*L* (nm)^b^	*W* (nm)^b^	*D* (nm)^b^	*eqW* (nm)	IARC classification*^,c^
HL-charged	Idarubicin	498	8.04, 10.04	+1	−0.94	1.273	0.903	0.363	0.573	N/A
	Imatinib	494	7.84, 12.69	+1	1.06	1.251	0.944	0.796	0.867	N/A
	Irinotecan	587	9.47, 11.71	+1	0.68	1.657	1.177	0.420	0.703	N/A
	Methotrexate	454	3.8, 4.8, 5.6	−2	−3.91	1.324	1.031	0.499	0.717	3
	Mitoxantrone	445	8.27, 9.36	+2	−2.35	1.926	0.926	0.182	0.410	2B
	Pemetrexed	427	2.43, 3.39	−2	−2.49	1.770	0.740	0.535	0.629	N/A
	Pentostatin	268	8.33, 13.06	+1	−3.81	0.966	0.588	0.331	0.441	N/A
	Pixantrone	325	9.52	+2	−3.53	1.320	0.925	0.182	0.410	N/A
	Raltitrexed	459	1.24, 3.72	−2	−3.45	1.399	1.068	0.752	0.896	N/A
	Topotecan	421	1.74, 8.00, 9.75	+1	−0.82	1.690	0.737	0.513	0.615	N/A
	Vinblastine	811	8.82, 10.87	+2	1.72	1.396	1.375	1.080	1.218	3
	Vincristine	825	8.66, 10.85	+2	0.55	1.743	1.140	0.921	1.024	3
	Vindesine	754	8.68, 11.34	+2	0.02	1.591	1.045	0.826	0.929	N/A
HL-neutral	Amsacrine	394	8.44, 10.82	0	2.29	1.226	0.995	0.698	0.833	2B
	Bicalutamide	430	12.00	0	2.53	1.066	1.058	1.058	1.058	N/A
	Cabazitaxel	836	−3.6, 11.96	0	5.32	1.444	1.381	0.901	1.115	N/A
	Carfilzomib	720	4.96, 11.91	0	4.43	1.636	1.285	0.721	0.962	N/A
	Docetaxel	808	−3, 11.9	0	3.54	1.197	1.123	0.786	0.939	N/A
	Flutamide	276	−3.7, 12.81	0	3.14	0.821	0.659	0.431	0.533	N/A
	Megestrol	343	−4.9, 17.83	0	3.48	3.480	0.753	0.587	0.665	N/A
	Mycophenolate mofetil	433	5.6, 8.5	0	2.99	1.594	0.925	0.502	0.681	N/A
	Paclitaxel	854	−1.2, 11.9	0	3.89	1.381	1.219	1.147	1.182	N/A
	Temsirolimus	1030	−2.9, 9.96	0	4.12	1.989	1.554	0.813	1.124	N/A
HB-charged	Mycophenolic acid	320	−4.1, 3.57	−1	2.57	1.011	0.871	0.182	0.398	N/A
	Tamoxifen	372	8.76	+1	3.98	1.487	1.208	0.555	0.819	1
	Trabectedin	762	7.2, 9.26	+1	2.86	1.341	0.921	0.680	0.791	N/A
	Vinorelbine	779	8.66, 10.87	+2	3.05	1.670	0.864	0.805	0.834	N/A

*MW* molecular weight, *W* molecular width, *L* molecular length, *D* molecular depth, *eqW* equivalent width, *1 carcinogenic to humans, *2A* probably carcinogenic to humans, *2B* possibly carcinogenic to humans, *3* not classifiable as to its carcinogenicity to humans, *N/A* no information available

^a^Acquired from Pubchem, Drugbank, and ChemSpider databases

^b^Determined through Chem3D desktop modelling program

^c^Listed in the International Agency for Research on Cancer (IARC 2022)

Regarding antineoplastic drugs’ IARC classification, it is noted that among the 54 compounds, only 15 are classified in terms of their carcinogenicity to humans (IARC 2022). Most of the target antineoplastic drugs are not classified due to the lack of toxicological data. From those classified, 6 antineoplastic drugs are carcinogenic to humans (busulfan, cyclophosphamide, etoposide, melphalan, tamoxifen, and thiotepa), 1 is probably carcinogenic (azacitidine), and 4 are possibly carcinogenic to humans (amsacrine, dacarbazine, mitomycin C, and mitoxantrone). Although nanofiltration and other removal technologies should be exploited and optimized for all legacy and emerging contaminants, special attention must be given to those that are proven to have any carcinogenic nature to humans because they can represent a threat to the environment and all living beings.

The average MW of the target pharmaceuticals is 446 ± 236 g/mol, being the lowest one 130 g/mol for fluorouracil and the highest one 1255 g/mol for dactinomycin. To facilitate the interpretation of the other pharmaceuticals’ chemical properties, they were then divided into four groups, according to their hydrophobicity and charge at neutral pH (pH = 7): hydrophilic and neutral compounds (HL-neutral), hydrophilic and charged compounds (HL-charged), hydrophobic and neutral compounds (HB-neutral), and hydrophobic and charged compounds (HB-charged) (Table 1). Of the 54 studied compounds, 10 of them are HB-neutral, 4 are HB-charged, in which 1 is negative and 3 are positive, 21 are HL-neutral, and 19 are HL-charged, being 6 of them negative and 13 positive.

The main theoretical rejection mechanism of a compound by a certain membrane depends on all properties compiled in Table 1, as well as membrane characteristics. It is expected that the rejection of a HL-neutral compound is mainly driven by steric hindrance effects (size exclusion mechanism), which means removals are highly dependent on the MW of the compounds and the molecular weight cut-off (MWCO) of the membrane, varying from low to high rejections. If HB-neutral, very low to low rejections are expected to be achieved also depending on the compound’s MW and membrane’s MWCO, due to hydrophobic interactions as the main rejection mechanism. On the other side, charged compounds are expected to be high to very high rejected by nanofiltration due to charge repulsion mechanism, regardless of their MW (Verliefde et al. 2007b). Still, there are many other factors that influence final rejections, such as the charge of the compound (negative or positive), operating conditions, membrane materials and configuration, characteristics of feed water, and possibility of membrane fouling (Suhalim et al. 2022; Verliefde et al. 2007b).

## Characterization and classification of the considered membranes

The selection of the membranes used for this study had into account the experimental assays that were already published regarding the rejection of antineoplastic drugs by nanofiltration: Desal 5DK (Cristóvão et al. 2022; Cristóvão et al. 2019; Wang et al. 2009), Desal HL (Verliefde et al. 2009; Verliefde et al. 2007a), Trisep TS-80 (Verliefde et al. 2009; Verliefde et al. 2007a), NF50 M10 (Kazner et al. 2008), and NF270 (Cristóvão et al. 2019). All the selected membranes have a polymer-base, with a poly(piper-azineamide) top layer composition (Petrinic et al. 2007), with acidic iso-electric points, and thus, they are negatively surface charged at neutral pH (Table 2). The main difference among the selected membranes stands on their MWCO, which varies between 100 and 400 Da, being the lowest one for Trisep TS-80 (100-200 Da) and the highest one for NF270 (200–400 Da) (Table 2).

**Table 2:** Characterization of the target membranes

	Desal 5DK	Desal HL	Trisep TS-80	DOW Filmtec NF270	X-FLOW NF50 M10
MWCO (Da)	150–300^a,b^	150–300^c^	100–200^d^	200–400^e^	200^f^
Membrane material	Polyamide thin-film composite				
Iso-electric point (IEP)	3.9^g^	3.9^c^	3.0^d^	3.2^b^	N/A
Membrane charge at neutral pH	Negative	Negative	Negative	Negative	N/A

*N/A* not available

^a^Causserand et al. (2005)

^b^Cristóvão et al. (2019)

^c^Verliefde et al. (2007a)

^d^NPD (2023)

^e^Ramdani et al. (2021)

^f^Kazner et al. (2008)

^g^Dalwani (2011)

Regarding the estimation of the rejections of antineoplastic drugs by each one of those membranes, the lower and the higher MWCO of each membrane were considered to account for the impact of this parameter on the final result: 100 and 200 Da for Trisep TS-80, 150 and 300 Da for Desal 5DK and Desal HL, and 200 and 400 Da for NF270. NF50 M10 was the only membrane for which only one MWCO dimension was used, 200 Da, since no MWCO range was found (Kazner et al. 2008). It is important to highlight that each membrane’s characteristic varies with the manufacturer, and with the operating conditions, which are not accounted for in this prediction analysis based on QSAR model.

## Quantitative structure-activity relationship (QSAR)

### QSAR methodology

A QSAR is a methodology that relates quantitively the activity of a set of compounds to chemical descriptors of the same compounds (Yangali-Quintanilla et al. 2010). In the study done by Yangali-Quintanilla and co-workers, a QSAR analysis was used to quantify the activity “compounds rejection by a membrane” in terms of organic-compound physical and chemical properties, membrane characteristics, and operating conditions (Yangali-Quintanilla et al. 2010). A total of twenty-one initial variables were considered including compound properties (MW, solubility, log*k*_*OW*_, log D, dipole moment, molar volume, *L*, *W*, *D*, and *eqW*), membrane characteristics (MWCO, pure water permeability, magnesium sulphate salt rejection, charge of the membrane as zeta potential, and hydrophobicity as contact angle), and operating conditions (operating pressure, cross-flow velocity, back diffusion mass transfer coefficient, flux, ratio of pure water permeation flux and back diffusion mass transfer coefficient and recovery) (Yangali-Quintanilla et al. 2010). Three methodologies aiming to simplify the several variables initially considered were tested: (i) principal component analysis, (ii) multiple linear regression, and (iii) principal component regression and partial least squares regression. Regarding the data range of operating conditions (pure water permeability, salt rejection, zeta potential, contact angle, and pressure), these were validated internally (106 rejection cases) and externally (from three different datasets obtained from other studies). The range of conditions tested and validated, as well as the ones used in the experimental studies, are mentioned in Table SI2. Thus, two final equations were obtained and validated by the authors, being both dependent on the log D and on the molecular size (*eqW*, *L*, and *D*) of the compound, having each equation one additional factor (the salt rejection or the MWCO of the membrane). Since salt rejection is a parameter that should be experimentally measured and is highly dependent on the operating conditions (Hagmeyer and Gimbel 1998), the equation dependent on the MWCO of the membrane was selected to estimate the rejections in the present study.

1 $$R=265.150\times eqW-117.356\times D+81.662\times L-5.229\times \log\ D-0.272\times MWCO-62.565$$

Being *R* the theoretical rejection (%), *eqW* the equivalent width (nm), *D* and *L* the size properties of each molecule (depth and length, in nm), and *MWCO* the molecular weight cut off (Da) of the nanofiltration membrane of interest, as previously explained.

Size parameters corresponding to the variables *L* and *eqW* may be high enough to result in rejection forecasts of more than 100%. This circumstance could also happen for rejection predictions of medium- to large-sized ionic compounds. Thus, according to Yangali-Quintanilla and co-workers, the rejection is considered 100% if the result of the equation is >100; if the result of the equation is below 100, then the rejection is the same value as the result (Yangali-Quintanilla et al. 2010).

### QSAR results for the studied antineoplastic drugs

**Fig. 1:**
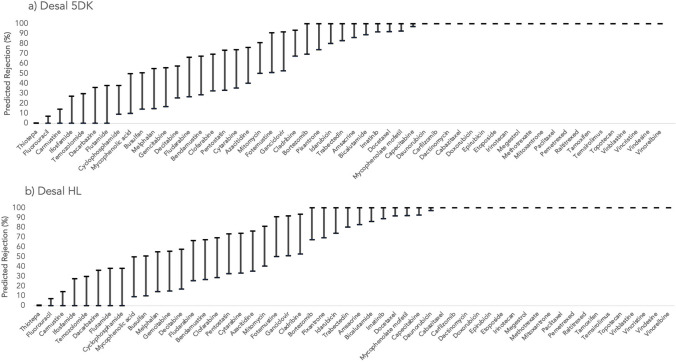
Predicted rejections (%) for the studied antineoplastic drugs using **a** Desal 5DK and **b** Desal HL membranes

**Fig. 2:**
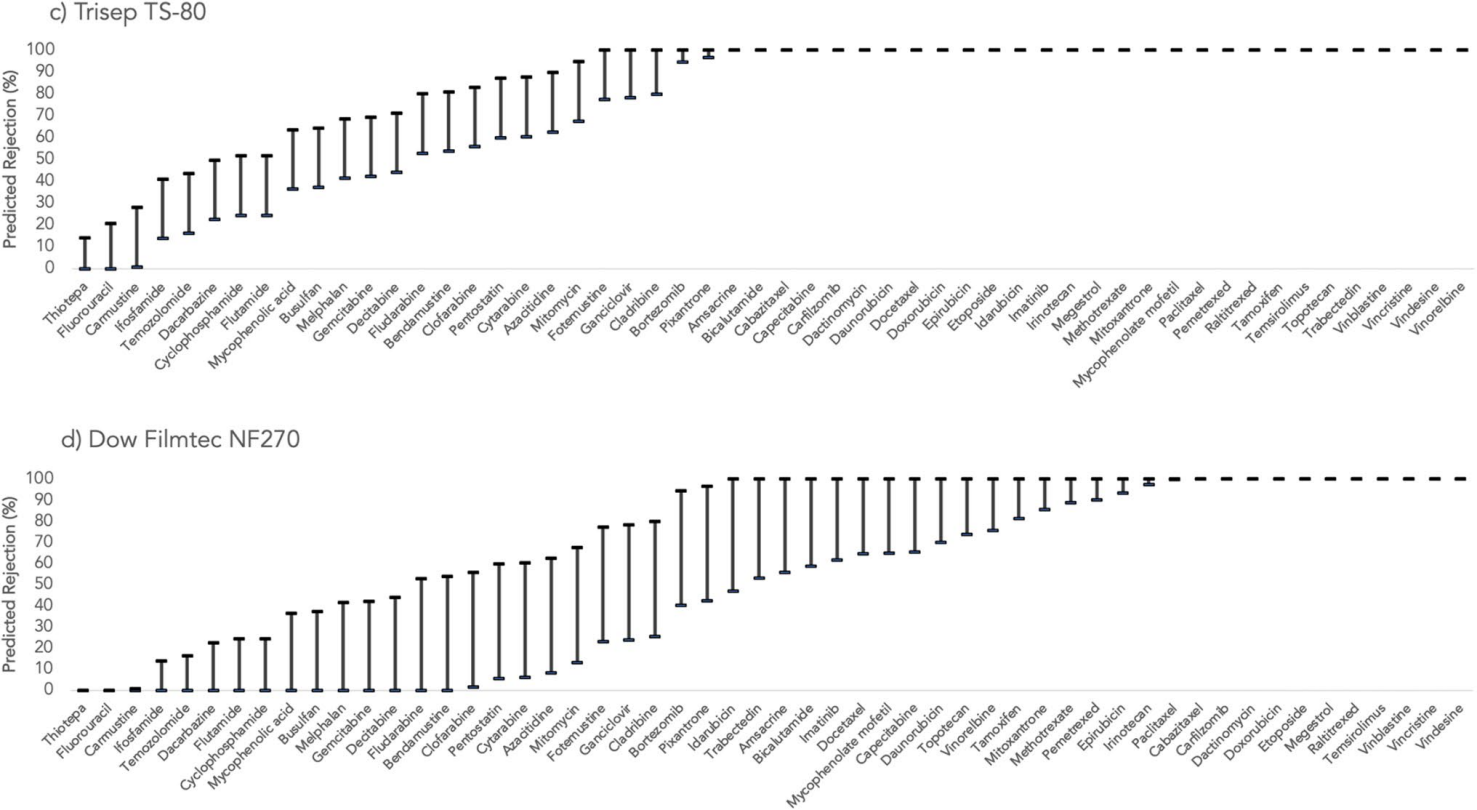
Predicted rejections (%) for the studied antineoplastic drugs using **c **Trisep TS-80 and **d **NF270 membranes

**Fig. 3:**
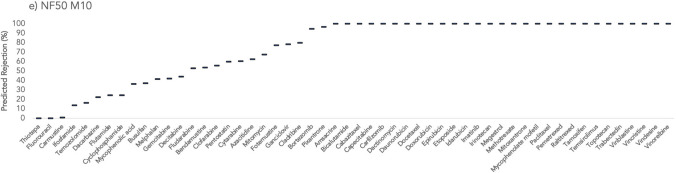
Predicted rejections (%) for the studied antineoplastic drugs using **e **NF50 M10 membrane

Figures 1, 2, and 3 represent the rejections predicted for the target antineoplastic drugs using the studied membranes (Desal 5DK, Desal HL, Trisep TS-80, NF270, and NF50 M10). The lowest and the highest values for each antineoplastic drug correspond to the rejections obtained from the highest and the lowest MWCO, respectively. Since NF50 M10 membrane only has one MWCO, then only one rejection value is presented for each compound considering that specific membrane.

A general analysis of the results indicates that the Trisep TS-80 membrane achieved the highest rejections, with an average of 80 ± 29%, for all antineoplastic drugs. This is explained by its lower MWCO (ranging from 100 to 200 Da). On the other hand, the NF270 membrane showed a wider range and lower overall rejections (an average rejection of 62 ± 40% for all antineoplastic drugs). NF270 membrane has a MWCO that could go up to 400 Da, which may justify these findings. From the 54 studied antineoplastic drugs, a rejection that can go up to 100% was predicted for 29 of them, regardless of the membrane used. Considering the worst-case scenario (if the membrane with the highest MWCO is used — 400 Da of NF270 membrane), 11 antineoplastic drugs would still be probably completely removed (carbazitaxel, carfilzomib, dactinomycin, doxorubicin, etoposide, megestrol, raltitrexed, temsirolimus, vinblastine, vincristine, and vindesine). Since all of these antineoplastic drugs have a MW higher than the MWCO of NF270, with the exception of megestrol (343 g/mol) (Table 1), their removal is likely governed by the size exclusion mechanism (sieving, steric effects). The reason for the complete rejection of megestrol might be related to its molecular length (*L* = 3.480 nm; Table 1), which is much higher than that of the remaining compounds length (average *L* = 1.28 nm). Even though megestrol is a HB-neutral compound, for which hydrophobic interactions are expected to be decisive in the main rejection mechanism (Gouveia et al. 2023), the size exclusion seems to be the main responsible for its complete removal in the QSAR prediction.

On the other side, there are two antineoplastic drugs for which negligible removals (≤21%) were predicted, even when the membrane with the lowest MWCO (100 Da of Trisep TS-80) was considered: fluorouracil and thiotepa. Once again, the size exclusion seems to be the governing factor in the rejection mechanism. Fluorouracil and thiotepa are both HL-neutral compounds, with MWs close to the lowest MWCO of Trisep TS-80 (130 g/mol for fluorouracil and 189 g/mol for thiotepa). Furthermore, these two antineoplastic drugs are of smaller size than others: *eqW* of 0.348 nm for fluorouracil, 0.306 nm for thiotepa, and 0.689 nm (average) for the others (Table 1). Between the considered membranes, if experimental NF assays are being conducted, the use of Trisep TS-80 is recommended due to the lower MWCO. However, incomplete removals were still predicted (up to 21%), and if a more efficient (or complete) elimination is required for fluorouracil and thiotepa, reverse osmosis could enhance the results due to its lower MWCO when compared to the nanofiltration’s (Rizzo et al. 2019).

Extra consideration should be taken in relation to antineoplastic drugs classified as carcinogenic to humans: busulfan, cyclophosphamide, etoposide, melphalan, tamoxifen, and thiotepa. Etoposide rejections were 100% for all target membranes, as previously mentioned. The rejection of busulfan, cyclophosphamide, and melphalan is highly dependent on the membrane used, since their MW (246 g/mol, 261 g/mol, and 305 g/mol, respectively) fall within the MWCO range of the target membranes. As HL-neutral compounds, their main rejection mechanism is expected to be by size exclusion, which corroborates the predictions. Their specific rejections vary between 0%, if a membrane with a higher MWCO is used (NF270), and 64% rejection for busulfan, 52% rejection for cyclophosphamide, and 69% rejection for melphalan when Trisep TS-80 is considered. Therefore, the Trisep TS-80 or another membrane with a lower MWCO are recommended for the removal of cyclophosphamide, busulfan, and melphalan, and should be therefore exploited in future experimental membrane studies on the matter. Nevertheless, up to 69% removals might not be considered a worthy accomplishment if these compounds are present in real streams that are going to be discharged to the environment (considering their carcinogenicity). Thus, additional or alternative removal treatments should be considered if a complete removal of these antineoplastic drugs is envisaged.

Regarding tamoxifen, a hydrophobic positively charged compound at neutral pH (HB-charged), high or very high rejections are expected to be achieved from negatively charged membranes due to charge repulsion effects (Verliefde et al. 2007b). In fact, predicted rejections for this antineoplastic drug varied from 81 to 100% for the target membranes, which is in accordance with the expectations.

Given the potential health risks associated with exposure to antineoplastic drugs, particularly those classified as carcinogenic to humans, it is crucial to implement experimental assays to assess/confirm the efficacy of nanofiltration in removing these compounds from wastewaters. While theoretical predictions can provide some insight into the expected rejections, there may be additional factors at play that can only be accurately evaluated through empirical testing.

### QSAR results: predicted versus measured rejections

Up to the authors’ knowledge, 7 original papers were found regarding nanofiltration processes applied to antineoplastic drugs (Cristóvão et al. 2022; Cristóvão et al. 2019; Gouveia et al. 2023; Kazner et al. 2008; Verliefde et al. 2009; Verliefde et al. 2007a; Wang et al. 2009). Only 13 out of the 54 target antineoplastic drugs were studied in the aforementioned original papers: bicalutamide, capecitabine, cyclophosphamide, cytarabine, etoposide, fluorouracil, flutamide, ifosfamide, megestrol, mycophenolate mofetil, mycophenolic acid, paclitaxel, and tamoxifen. Desal 5DK was the most studied membrane (4 out of 7 studies), and it was tested for bicalutamide, capecitabine, cyclophosphamide, etoposide, flutamide, ifosfamide, megestrol, mycophenolate mofetil, mycophenolic acid, paclitaxel, and tamoxifen (Cristóvão et al. 2022; Cristóvão et al. 2019; Gouveia et al. 2023; Wang et al. 2009). Desal 5 DK’ highly hydrophilic character, relatively low MWCO and high-water permeability, may be among the reasons for its higher popularity, when compared to other nanofiltration membranes for the removal of pharmaceuticals in general (Oliveira et al. 2022). Up to the authors’ knowledge, NF50 M10 was only used in one study, for the rejection of cytarabine and fluorouracil (Kazner et al. 2008). The low usage frequency of NF50 M10 membrane may be attributed to the membranes’ capillary/hollow fiber configuration, which contributes to its high costs, challenging production process, and the associated difficulties with environmental legislation due to the high chemical usage (Jonkers et al. 2023).

**Fig. 4:**
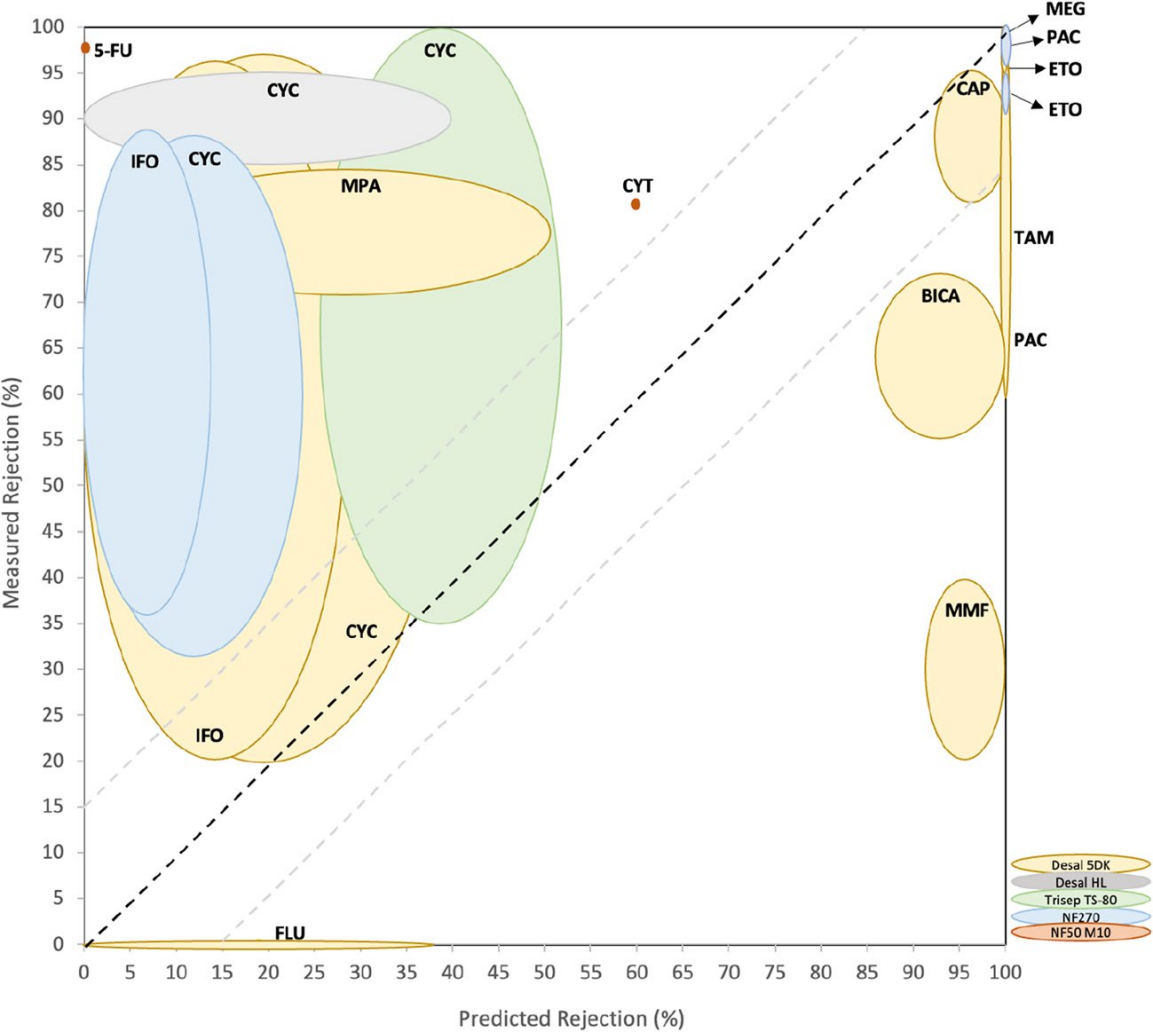
Measured *versus* predicted rejections obtained for the studied antineoplastic drugs and membranes. BICA, bicalutamide; CAP, capecitabine; CYC, cyclophosphamide; CYT, cytarabine; ETO, etoposide; 5-FU, fluorouracil; FLU, flutamide; IFO, ifosfamide; MEG, megestrol; MMF, mycophenolate mofetil; MPA, mycophenolic acid; PAC, paclitaxel; TAM, tamoxifen

The rejections measured experimentally, found in the literature, are compiled in Table SI1. The same values were then compared with the rejections predicted in the present work through QSAR model, and Fig. 4 was drawn.

Looking at Fig. 4, the more the bullets fall in the *y* = *x* line, the better the correlation between experimental and theoretical data, i.e., the more precise and accurate the predictive model is.

A general overview of the results presented in Fig. 4 indicates that (i) cyclophosphamide and ifosfamide are the compounds for which a higher range of rejections was measured experimentally (they are also the antineoplastic drugs for which more experimental assays were conducted) and (ii) some of the measured rejections are in accordance with the ones predicted, not deviating more than 15% from each other, as can be seen for capecitabine (Desal 5DK), etoposide (for Desal 5DK and NF270), paclitaxel (for Desal 5DK and NF270), megestrol (Desal 5DK), flutamide (Desal 5DK), tamoxifen (Desal 5DK), cyclophosphamide (Desal 5DK and Trisep TS-80), and ifosfamide (Desal 5DK). However, some exceptions can be noticed as happens with bicalutamide (Desal 5DK), mycophenolic acid (Desal 5DK), mycophenolate mofetil (Desal 5DK), cytarabine (NF50 M10), fluorouracil (NF50 M10), cyclophosphamide (Desal HL and NF270), and ifosfamide (NF270), for which deviations higher than 15% between measured and predicted rejections were noticed.

#### HL-neutral antineoplastic drugs

As mentioned in the previous section, HL-neutral compounds are expected to be rejected by size exclusion mechanism. From the antineoplastic drugs studied experimentally with this classification (capecitabine, cyclophosphamide, etoposide, fluorouracil, and ifosfamide), it can be concluded that most of the predicted rejections are close to the experimental results (or that there are at least some experimental results for these compounds that are less than 15% different from the predicted ones). Exceptions were verified for fluorouracil considering NF50 M10 membrane, and cyclophosphamide and ifosfamide with NF270 membrane (Fig. 4).

Kazner and co-workers measured a rejection of 97% for fluorouracil, in a capillary NF50 M10 nanofiltration unit using real a wastewater as matrix (Kazner et al. 2008) — more details presented in Table SI1. Predictions using QSAR model revealed a predicted rejection of 0% for this compound, which is highly far from the experimental results (Fig. 4). The fluorouracil’s MW (130 g/mol) is lower than membranes’ MWCO (200 Da), and this may justify the findings. However, despite size exclusion being the theoretical preferred mechanism for fluorouracil’s rejection, there are several parameters that may improve or decrease real rejections (Dalwani 2011). The membrane fouling, for example, may have led to higher rejections than expected in Kazner and co-workers study. Other operating conditions, such as medium pH and matrix constituents, may also have an impact in the final results. More experimental assays and alternative degradation techniques should be conducted regarding fluorouracil’s removal from wastewaters.

Cyclophosphamide is the most studied antineoplastic drug in terms of its rejection by nanofiltration membranes. As mentioned in “QSAR results for the studied antineoplastic drugs” of the preset study, the QSAR prediction led to rejections varying from negligible (0%) that can be achieved for Desal 5DK, Desal HL, and NF270 to 52% for Trisep TS-80. Experimental data regarding cyclophosphamide removal from nanofiltration membranes is highly variable, being reported rejections varying from 20 to 97% for Desal 5DK (Cristóvão et al. 2022; Cristóvão et al. 2019; Gouveia et al. 2023; Wang et al. 2009), from 85 to 95% for Desal HL (Verliefde et al. 2007a), from 31 to 87% for NF270 (Cristóvão et al. 2019), and from 35 to 100% for Trisep TS-80 (Verliefde et al. 2009). Detailed information is presented in Table SI1. The same trend was verified for ifosfamide, which showed rejections varying from 20% (Gouveia et al. 2023) to >96% (Cristóvão et al. 2022), both works using a Desal 5DK membrane. The high variability in cyclophosphamide and ifosfamide results may be justified by their MWs (261 g/mol), which are within the MWCO range of the studied membranes (100–400 Da). This means that the lower the pore size of the membrane, the higher the rejections obtained for these compounds. Still, QSAR model predicted negligible removals for both compounds in certain circumstances (for example when NF270 membrane is considered), which was not verified experimentally, since all the measured rejections for these compounds are >20%. For example, Cristóvão and co-workers achieved 45.3% rejection for cyclophosphamide and 43.8% rejection for ifosfamide using NF270 membrane in wastewaters (Cristóvão et al. 2019) (Table SI1). The lower length (*L*) of cyclophosphamide (0.792 nm) and ifosfamide (0.680 nm) molecules, when compared to the remaining antineoplastics’ *L* (average of 1.275 nm), may let both molecules pass at a certain extent through the membrane pores, preventing final rejections to be negligible as predicted.

Only one study experimentally tested etoposide’s rejection by nanofiltration, from three different matrices (laboratory grade water, synthetic urine, and wastewaters), using Desal 5DK and NF270 membranes (Cristóvão et al. 2019) (Table SI1). Cristóvão and co-workers concluded that etoposide was mainly removed by size exclusion mechanism, being achieved rejections >95% using Desal 5DK and from 91% to >95% using NF270. Predictions obtained through QSAR model expected etoposide to be 100% rejected from both membranes, neighboring the experimental results (Fig. 4).

The only membrane experimentally tested for capecitabine rejection was Desal 5DK, in a pilot-scale nanofiltration system operating with real wastewaters (Cristóvão et al. 2022; Gouveia et al. 2023). Experimental rejections varying from 82 to >96%, alike the predicted rejections from the QSAR model, which varied from 93 to 100% (Fig. 4).

#### HB-neutral antineoplastic drugs

As previously referred, very low to low rejections are expected to be achieved for HB-neutral compounds, depending on their MW and membranes’ MWCO, due to hydrophobic interactions (Verliefde et al. 2007a). Hydrophobic interactions between solute and membrane can cause adsorption of the compound on the membrane surface and in the membrane pores. The expected amount of adsorption on the membrane increases with a chemical’s hydrophobicity. High initial rejections may result from initial hydrophobic molecule adsorption, which eventually drops to an equilibrium concentration when the breakthrough is noticed. The compound’s MW will have an impact on the equilibrium rejection value (Verliefde et al. 2007b). From the 10 HB-neutral antineoplastic drugs considered in this study, only the rejections of 5 of them were experimentally obtained: bicalutamide, flutamide, megestrol, mycophenolate mofetil, and paclitaxel. Table SI1 presents the results of each study. The experimental rejections of megestrol, paclitaxel, and flutamide were relatively close to the perditions. An opposite trend was verified for bicalutamide and mycophenolate mofetil, with variations >15% between measured and predicted rejections (Fig. 4).

Megestrol’s predicted and measured rejections are very similar to each other when Desal 5DK membrane is considered (97.9–98.7% measured *vs* 100% predicted) (Fig. 4). Experimental assays were performed by Gouveia and co-workers, who tested the removal of megestrol from wastewaters in a pilot-scale nanofiltration system with a spiral wound Desal 5DK nanofiltration membrane (Gouveia et al. 2023).

Paclitaxel rejections by two nanofiltration membranes (Desal 5DK and NF270) were investigated considering three different matrices (laboratory grade water, synthetic urine, and wastewaters) (Cristóvão et al. 2019; Gouveia et al. 2023). Desal 5DK led to rejections from 59 to >95% (Cristóvão et al. 2019; Gouveia et al. 2023), while NF270 to rejections >95% (Cristóvão et al. 2019) (Table SI1). Predicted rejection for paclitaxel from QSAR model is >99.9%, both for Desal 5DK and NF270 membranes. The high MW of paclitaxel (854 g/mol) may certainly interfere with final rejections, both experimental and predicted ones.

Regarding flutamide, low rejections were predicted from the QSAR model (up to 38%) using the Desal 5DK membrane. This was confirmed experimentally by Gouveia and co-workers (Gouveia et al. 2023), who measured negligible rejections for this compound using the same membrane (Table SI1). As expected, flutamide’s hydrophobic nature (HB-neutral), together with the fact of its MW (261 g/mol) being within membranes’ MWCO (150–300 Da), corroborates flutamides’ very low rejections.

Bicalutamide rejection by nanofiltration has only been reported in one study, where Desal 5DK was used in a pilot-scale equipment with real wastewater (Gouveia et al. 2023). Rejections ranging from 55 to 73% were obtained in this study, which are lower than the predicted rejections by the QSAR model (86–100%) (Fig. 4). The higher rejection predicted by the QSAR model may be justified by the high MW of bicalutamide (430 g/mol) when compared to membranes’ MWCO (150–300 Da). However, the experimental assays seem to suggest that the hydrophobicity of bicalutamide may negatively impact the rejections (i.e., lower rejection are obtained experimentally).

Looking at mycophenolate mofetil, only one study measured its retention on a nanofiltration membrane (Desal 5DK), in a pilot-scale unit using real wastewaters (Gouveia et al. 2023); rejections varying from 20 to 40% were obtained by the group (more details presented in Table SI1). Much higher rejections (92–100%) would be expected, according to the QSAR model, probably due to the fact that its MW (433 g/mol) is much higher than the MWCO of membranes (150–300 Da). The hydrophobic character of mycophenolate mofetil might also have influenced experimental results, which highlights a possible weakness of the QSAR methodology for hydrophobic compounds.

#### HL- and HB-charged compounds

As previously seen, charged compounds are expected to be high or very high rejected by nanofiltration due to charge repulsion mechanism, as a result of their inability to approach negatively charged membranes (electrostatic repulsion), which prevents them from passing through them, regardless of their MW (Verliefde et al. 2007b). On the other side, the rejection of pharmaceuticals with positive charges may be lower. This phenomenon is attributed to attractive forces that draw the solute towards the membrane. The greater the solute’s hydrophobicity, the higher its tendency to adsorb onto the membrane’s surface or pores (Verliefde et al. 2007b). From the 23 charged antineoplastic drugs considered (4 HB-charged and 19 HL-charged), only 3 of them are simultaneously charged and studied experimentally: cytarabine, mycophenolic acid, and tamoxifen. From these compounds, only tamoxifen rejection was well predicted by the QSAR model.

Regarding tamoxifen (positively charged) and mycophenolic acid (negatively charged), only one study measured experimentally their rejections by a nanofiltration membrane (Desal 5DK), in a pilot-scale unit using real wastewaters (Gouveia et al. 2023). Both compounds showed relatively high rejections: 72–92% for tamoxifen and 71–85% for mycophenolic acid (Gouveia et al. 2023) (Table SI1). The predicted rejection for tamoxifen using the Desal 5DK membrane was 100%, which closely matched the experimental results and is aligned with the expected rejections. However, due to tamoxifen’s positive charge, there is a possibility of achieving higher rejections if short-time frames or small volumes of water are used, as these factors have been found to overestimate the rejections of positively charged and hydrophobic solutes (Chang et al. 2002; Kimura et al. 2003). Therefore, it is crucial to conduct experiments lasting more than 24 h to ensure the measurement of rejections at equilibrium. Regarding mycophenolic acid, QSAR prediction (9–50% rejection) is not in accordance with measured results (71–85% rejection). The fact that mycophenolic acid’s MW (320 g/mol) is in the upper range of membranes’ MWCO (150–300 Da) certainly led to the low/moderate predicted removals. However, experimental assays confirmed mycophenolic acid’s negative charge is repulsed from the negatively charged membrane, conferring higher removals through charge repulsion effects.

Cytarabine rejection by nanofiltration was only studied using NF50 M10 membrane by Kazner et al. (2008). They achieved a rejection of 81% for cytarabine using real wastewater as matrix (Kazner et al. 2008). The QSAR model predictions indicate a 60% rejection rate for cytarabine, which deviates from the experimental results by over 15%. As happens with mycophenolic acid, it can be seen that QSAR predictions may be limited when considering charged compounds, especially if their MW are of the same range of MWCO of membranes.

## Conclusions

In this study, a general quantitative structure-activity relationship (QSAR) model was used to predict rejections based on an integral approach. This QSAR model contemplates membrane characteristics, filtration operating conditions, and physicochemical compound properties and was developed and validated by Yangali-Quintanilla et al. (2010). The retention of 54 antineoplastic drugs was predicted by 5 different polyamide nanofiltration membranes (Desal 5DK, Desal HL, Trisep TS-80, NF270, and NF50 M10), using the same QSAR model.

Out of the 54 studied antineoplastic drugs, 29 were predicted to have a rejection that could go up to 100%, independent of the membrane used. Nonetheless, there were 2 antineoplastic drugs, fluorouracil and thiotepa, for which negligible removals were obtained (<21%) due to their small molecular size and low MW. The QSAR model exhibited reliable accuracy for HL-neutral compounds, with some exceptions potentially attributed to operating conditions (as seen for fluorouracil). However, the predicted rejections of hydrophobic (HB) and charged (HL- and HB-charged) compounds by nanofiltration membranes were proved to be quite limited, potentially resulting in inaccurate predictions.

This model may also allow an easier prioritization of the most important antineoplastic drugs to be included in different degradation assays (e.g., advanced oxidation processes), especially if toxic and predicted not to be removed by membrane-based technologies, as in the case of thiotepa.

Knowledge of nanofiltration membrane characteristics is crucial for accurate separation behavior predictions. While the QSAR model offers valuable preliminary estimations, it should not replace experimental assays.

### Supplementary information

ESM 1 (DOCX 34 kb)
