# Supplementary material for: Theoretical rejection of fifty-four antineoplastic drugs by different nanofiltration membranes
Source: Environ Sci Pollut Res Int. 2023 Sep 19;30(48):106099–111. doi: 10.1007/s11356-023-29830-w (PMC10579118; doi:10.1007/s11356-023-29830-w)
Supplement: Supplementary file 1 — (DOCX 34 kb) [file 11356_2023_29830_MOESM1_ESM.docx]

**Theoretical rejection of fifty-four antineoplastic drugs by different nanofiltration membranes**

Teresa I.A. Gouveia^a,b^, Arminda Alves^a,b^, Mónica S.F. Santos^a,b,c,d *^

^a^ LEPABE – Laboratory for Process, Environmental, Biotechnology and Energy Engineering, Faculty of Engineering, University of Porto, R. Dr. Roberto Frias, 4200-465 Porto, Portugal

^b^ALiCE – Associate Laboratory in Chemical Engineering, Faculty of Engineering, University of Porto, Rua Dr. Roberto Frias, 4200-465 Porto, Portugal

^c^ EPIUnit - Institute of Public Health, University of Porto, Rua das Taipas, n° 135, 4050-600 Porto, Portugal

^d^ ITR - Laboratory for Integrative and Translational Research in Population Health, University of Porto, Rua das Taipas, n° 135, 4050-600 Porto, Portugal

[*monica.santos@ispup.up.pt](mailto:*monica.santos@ispup.up.pt)

**Table SI1**- Nanofiltration applied for antineoplastic drugs’ removal from waters and wastewaters (literature review).

| **Antineoplastic drug** | **Matrix** | **Membrane** | **Operating conditions** | **Rejections (%)** | **Ref.** |
| --- | --- | --- | --- | --- | --- |
| Cyclophosphamide | Surface water | Trisep TS-80 | C_0_ = 2 μg/L;  P_TM_ = 5 bar;  10% feed water recovery | ~100% | Verliefde *et al.* (2007) |
| Cyclophosphamide | Surface water | Desal HL | C_0_ = 2 μg/L;  P_TM_ = 5 bar;  10% feed water recovery | ~95% | Verliefde *et al.* (2007) |
| Cyclophosphamide | Surface water | Trisep TS-80 | C_0_ = 100 μg/L;  P_TM_ = 5 bar;  80% feed water recovery | ~35% | Verliefde *et al.* (2007) |
| fluorouracil, cytarabine | WWTP effluent | NF50 M10 | C_0_ = 2.5 μg/L for 5-FU and 1.6 μg/L for CYT;  P_TM_ = 2.5 bar;  75% feed water recovery | 97.2% for fluorouracil and 80.8% for cytarabine | Kazner *et al.* (2008) |
| Cyclophosphamide | Ultrapure water  (+5 mM NaCl) | Desal HL | C_0_ = 2 μg/L;  P_TM_ = 5 bar | ~85% | Verliefde *et al.* (2009) |
| Cyclophosphamide | Ultrapure water  (+5 mM NaCl) | Trisep TS-80 | C_0_ = 2 μg/L;  P_TM_ = 5 bar | ~88% | Verliefde *et al.* (2009) |
| Cyclophosphamide | Ultrapure water | Desal 5DK | C_0_ = 10 to 600 μg/L;  P_TM_ = 20 bar;  Vp = 100 mL | ~40% | Wang *et al.* (2009) |
| Cyclophosphamide | Ultrapure water | Desal 5DK | C_0_ = 10 to 600 μg/L;  P_TM_ = 20 bar;  Vp = 200 mL | ~20% | Wang *et al.* (2009) |
| Cyclophosphamide | Effluent from MBR | Desal 5DK | C_0_ = 10 μg/L;  P_TM_ = 20 bar;  Vp = 100 mL | ~55% | Wang *et al.* (2009) |
| Cyclophosphamide | Effluent from MBR | Desal 5DK | C_0_ = 10 μg/L;  P_TM_ = 20 bar;  Vp = 200 mL | ~60% | Wang *et al.* (2009) |
| Paclitaxel, etoposide, cyclophosphamide, ifosfamide | Laboratory grade water | Desal 5DK | C_0_ = 500 µg/L;  P_TM_ = 10 bar;  Vp = 200 mL | >95% for paclitaxel; 97.7% for etoposide; 86.2% for cyclophosphamide and 84.8% for ifosfamide | Cristóvão *et al.* (2019) |
| Paclitaxel, etoposide, cyclophosphamide, ifosfamide | Laboratory grade water | NF270 | C_0_ = 500 µg/L;  P_TM_ = 10 bar;  Vp = 200 mL | >95% for paclitaxel; 93.1% for etoposide; 31-87% for cyclophosphamide and 36-88% for ifosfamide | Cristóvão *et al.* (2019) |
| Paclitaxel, etoposide, cyclophosphamide, ifosfamide | Synthetic urine | Desal 5DK | C_0_ = 500 µg/L;  P_TM_ = 10 bar;  Vp = 200 mL | >95% for paclitaxel; >95% for etoposide; 96.6% for cyclophosphamide and 96.3% for ifosfamide | Cristóvão *et al.* (2019) |
| Paclitaxel, etoposide, cyclophosphamide, ifosfamide | Synthetic urine | NF270 | C_0_ = 500 µg/L;  P_TM_ = 10 bar;  Vp = 200 mL | >95% for paclitaxel; >95% for etoposide; 81.1% for cyclophosphamide and 82.5% for ifosfamide | Cristóvão *et al.* (2019) |
| Paclitaxel, etoposide, cyclophosphamide, ifosfamide | WWTP effluent | Desal 5DK | C_0_ = 500 µg/L;  P_TM_ = 10 bar;  Vp = 200 mL | 99.9% for paclitaxel; 98.7% for etoposide; 90.4% for cyclophosphamide and 88.8% for ifosfamide | Cristóvão *et al.* (2019) |
| Paclitaxel, etoposide, cyclophosphamide, ifosfamide | WWTP effluent | NF270 | C_0_ = 500 µg/L;  P_TM_ = 10 bar;  Vp = 200 mL | 99.9% for paclitaxel; 91.0% for etoposide; 45.3% for cyclophosphamide and 43.8% for ifosfamide | Cristóvão *et al.* (2019) |
| Capecitabine, cyclophosphamide, ifosfamide | Domestic WWTP | Desal 5DK | C_0_ = 8-60 ng/L;  P_TM_ = 6 bar;  Vp = 1000 L | >96% for all compounds (not detected) | Cristóvão *et al.* (2022) |
| Bicalutamide, capecitabine, cyclophosphamide, flutamide, ifosfamide, megestrol, mycophenolate mofetil, mycophenolic acid, paclitaxel, tamoxifen | Domestic WWTP | Desal 5DK | C_0_ = 0.1 -80 ng/L; 70% water recovery; Vp = 1000 L | 55-73% for bicalutamide; 82-96% for capecitabine; >92% or <43% for cyclophosphamide; 0% for flutamide; 20-40% for ifosfamide; 97.9-98.7% for megestrol; 20-40% for mycophenolate mofetil; 71-85% for mycophenolic acid; 59-71% for paclitaxel and 72-92% for tamoxifen | Gouveia *et al.* (2023) |

**Table SI2** - Data range of operating conditions: validation of the QSAR methodology *versus* experimental studies using antineoplastic drugs.

|  | **QSAR model**  **(min – max)** | **Experimental**  **(min – max)** | **Reference** |
| --- | --- | --- | --- |
| Pure water permeability  (PWP, L/m^2^/h)/bar) | 3.58 – 9.29 | 2.8 – 11.05 | Cristóvão *et al.* (2019) |
| Salt Rejection (SR) | 96 – 99 | 98 – 99 | Verliefde *et al.* (2009); Verliefde *et al.* (2007); Wang *et al.* (2009) |
| Zeta potential (ZP, MV) | -48.04 – (-10.78) | -30 – (-10) | Verliefde *et al.* (2009); Verliefde *et al.* (2007) |
| Contact angle | 39.3 – 58.0 | 35 – 48 | Verliefde *et al.* (2009); Verliefde *et al.* (2007) |
| Pressure (P, bar) | 2.76 – 4.83 | 2.5 – 10 | Cristóvão *et al.* (2019); Kazner *et al.* (2008) |

**References**

Cristóvão, M. B., Bernardo, J., Bento-Silva, A., Ressureição, M., Bronze, M. R., Crespo, J. G. and Pereira, V. J. 2022. Treatment of anticancer drugs in a real wastewater effluent using nanofiltration: a pilot scale study. Separation and Purification Technology, 120565. <https://doi.org/10.1016/j.seppur.2022.120565>.

Cristóvão, M. B., Torrejais, J., Janssens, R., Luis, P., Van der Bruggen, B., Dubey, K. K., . . . Pereira, V. J. 2019. Treatment of anticancer drugs in hospital and wastewater effluents using nanofiltration. Separation and Purification Technology 224, 273-280. <https://doi.org/10.1016/j.seppur.2019.05.016>.

Gouveia, T.I.A., Cristóvão, M.B., Pereira, V.J., J.G., Crespo, Alves, A., Ribeiro, A.R., . . . Santos, M.S.F. 2023. Antineoplastic drugs in urban wastewater: occurrence, nanofiltration treatment and toxicity screening. Environmental Pollution 121944. <https://doi.org/10.1016/j.envpol.2023.121944>.

Kazner, C., Lehnberg, K., Kovalova, L., Wintgens, T., Melin, T., Hollender, J. and Dott, W. 2008. Removal of endocrine disruptors and cytostatics from effluent by nanofiltration in combination with adsorption on powdered activated carbon. Water Science & Technology 58(8), 1699-1706. <https://doi.org/10.2166/wst.2008.542>.

Verliefde, A. R. D., Cornelissen, E. R., Heijman, S. G. J., Petrinic, I., Luxbacher, T., Amy, G. L., . . . van Dijk, J. C. 2009. Influence of membrane fouling by (pretreated) surface water on rejection of pharmaceutically active compounds (PhACs) by nanofiltration membranes. Journal of Membrane Science 330(1), 90-103. <https://doi.org/10.1016/j.memsci.2008.12.039>.

Verliefde, A. R. D., Heijman, S. G. J., Cornelissen, E. R., Amy, G., Van der Bruggen, B. and van Dijk, J. C. 2007. Influence of electrostatic interactions on the rejection with NF and assessment of the removal efficiency during NF/GAC treatment of pharmaceutically active compounds in surface water. Water Research 41(15), 3227-3240. <https://doi.org/10.1016/j.watres.2007.05.022>.

Wang, L., Albasi, C., Faucet-Marquis, V., Pfohl-Leszkowicz, A., Dorandeu, C., Marion, B. and Causserand, C. 2009. Cyclophosphamide removal from water by nanofiltration and reverse osmosis membrane. Water Research 43(17), 4115-4122. <https://doi.org/10.1016/j.watres.2009.06.007>.
